# Associations of Country-Specific and Sociodemographic Factors With Self-Reported COVID-19–Related Symptoms: Multivariable Analysis of Data From the CoronaCheck Mobile Health Platform

**DOI:** 10.2196/40958

**Published:** 2023-02-03

**Authors:** Elke Humer, Thomas Keil, Carolin Stupp, Winfried Schlee, Manfred Wildner, Peter Heuschmann, Michael Winter, Thomas Probst, Rüdiger Pryss

**Affiliations:** 1 Department for Psychosomatic Medicine and Psychotherapy University for Continuing Education Krems Krems Austria; 2 Institute of Clinical Epidemiology and Biometry University of Würzburg Würzburg Germany; 3 State Institute of Health Bavarian Health and Food Safety Authority Erlangen Germany; 4 Institute of Social Medicine, Epidemiology and Health Economics Charité – Universitätsmedizin Berlin Berlin Germany; 5 Department of Psychiatry and Psychotherapy University of Regensburg Regensburg Germany; 6 Eastern Switzerland University of Applied Sciences St Gallen Switzerland; 7 Pettenkofer School of Public Health University of Munich Munich Germany; 8 Clinical Trial Center Würzburg University Hospital Würzburg Würzburg Germany

**Keywords:** COVID-19, COVID-19 symptoms, gender, India, South Africa, Germany, symptoms, app, information, English, sociodemographic, weakness, muscle pain, pain, age, education

## Abstract

**Background:**

The COVID-19 symptom-monitoring apps provide direct feedback to users about the suspected risk of infection with SARS-CoV-2 and advice on how to proceed to prevent the spread of the virus. We have developed the CoronaCheck mobile health (mHealth) platform, the first free app that provides easy access to valid information about the risk of infection with SARS-CoV-2 in English and German. Previous studies have suggested that the clinical characteristics of individuals infected with SARS-CoV-2 vary by age, gender, and viral variant; however, potential differences between countries have not been adequately studied.

**Objective:**

The aim of this study is to describe the characteristics of the users of the CoronaCheck mHealth platform and to determine country-specific and sociodemographic associations of COVID-19–related symptoms and previous contacts with individuals infected with COVID-19.

**Methods:**

Between April 8, 2020, and February 3, 2022, data on sociodemographic characteristics, symptoms, and reports of previous close contacts with individuals infected with COVID-19 were collected from CoronaCheck users in different countries. Multivariable logistic regression analyses were performed to examine whether self-reports of COVID-19–related symptoms and recent contact with a person infected with COVID-19 differed between countries (Germany, India, South Africa), gender identities, age groups, education, and calendar year.

**Results:**

Most app users (N=23,179) were from Germany (n=8116, 35.0%), India (n=6622, 28.6%), and South Africa (n=3705, 16.0%). Most data were collected in 2020 (n=19,723, 85.1%). In addition, 64% (n=14,842) of the users were male, 52.1% (n=12,077) were ≥30 years old, and 38.6% (n=8953) had an education level of more than 11 years of schooling. Headache, muscle pain, fever, loss of smell, loss of taste, and previous contacts with individuals infected with COVID-19 were reported more frequently by users in India (adjusted odds ratios [aORs] 1.3-8.3, 95% CI 1.2-9.2) and South Africa (aORs 1.1-2.6, 95% CI 1.0-3.0) than those in Germany. Cough, general weakness, sore throat, and shortness of breath were more frequently reported in India (aORs 1.3-2.6, 95% CI 1.2-2.9) compared to Germany. Gender-diverse users reported symptoms and contacts with confirmed COVID-19 cases more often compared to male users.

**Conclusions:**

Patterns of self-reported COVID-19–related symptoms and awareness of a previous contact with individuals infected with COVID-19 seemed to differ between India, South Africa, and Germany, as well as by gender identity in these countries. Viral symptom–collecting apps, such as the CoronaCheck mHealth platform, may be promising tools for pandemics to support appropriate assessments. Future mHealth research on country-specific differences during a pandemic should aim to recruit representative samples.

## Introduction

The emergence of COVID-19, caused by SARS-CoV-2 infection, has led to a major ongoing public health crisis worldwide [1]. By April 2022, the COVID-19 pandemic had been associated with more than 6 million deaths out of more than 500 million confirmed cases worldwide [2]. To contain the spread of the virus, various prevention strategies, such as lockdowns, quarantine, and stringent hygiene practices, have been adopted in countries worldwide [3]. The transmission of SARS-CoV-2 occurs primarily through contact with infected individuals via respiratory droplets and aerosols [4,5]. Symptoms vary widely, with fever, cough, and fatigue among the most common [6,7], while loss of smell and loss of taste were described as the most prominent COVID-19 symptoms until 2021, before the Omicron variant became prevalent [7-9]. Symptoms may appear after an incubation period of 2-18 days after contact with the virus [3,10]. Viral shedding may occur 3 days before the onset of symptoms, and more than 50% of SARS-CoV-2 transmissions are reported to occur in asymptomatic infected individuals [11]. Therefore, early identification and isolation of individuals infected with COVID-19 is particularly important to reduce transmission [12,13]. To effectively prevent and manage COVID-19 cases (eg, by detecting clusters) at the population level, various mobile health (mHealth) apps have been developed [3,14]. Symptom-monitoring apps aim to determine whether the user may be infected with SARS-CoV-2 by asking a series of screening questions that include symptoms, such as fever, cough, and pain, and by documenting potential contacts with infected individuals. These apps aim to provide the user with information about the potential risk of being infected and to give advice about further behavior, such as avoiding physical contact with others or wearing a face mask to reduce the spread [3]. Some evidence exists suggesting that the reported clinical characteristics of individuals infected with SARS-CoV-2 vary by age, gender, variant of the virus, and type of reporting system (eg, self-reports vs clinician reported) [7,11,15-17]; however, possible differences between countries have not yet been sufficiently investigated. Since health systems operate differently worldwide (eg, centralized vs decentralized) and have responded differently to the pandemic itself, such country-specific comparisons are important [18]. Official COVID-19 data often do not reflect differences between countries, because they are subject to various uncertainties, such as small numbers of COVID-19 tests and shortcomings in the monitoring systems of countries with limited resources. Therefore, studies comparing cross-national cases run the risk of arriving at incorrect conclusions [19].

In the beginning, the CoronaCheck app was intended to enable symptom monitoring to offer quick advice to those affected. This advice needed to follow the official recommendations of the Robert Koch Institute as the National German Public Health Institute so that those affected could assess their symptoms to the best of the current knowledge and to complement the official support hotlines. Another goal was to provide certain segments of the population with easier access to information than through a telephone hotline. These included adolescents, people with hearing problems who may not fully understand the telephone voice, and migrants or tourists without sufficient language skills for a hotline in the local language.

CoronaCheck is an open-science mHealth platform with 109,603 installations and 88,537 completed questionnaires as of February 3, 2022. The app was developed in the first months of the COVID-19 pandemic in Germany in collaboration with university partners from Bavaria and software companies. The app is based on the TrackYourHealth platform [20,21] and is guided by the information and official recommendations of health authorities. The goal is to provide users with quick and easy-to-perform symptom screening and documentation of contact information to identify their risk of infection with SARS-CoV-2 [22]. CoronaCheck adheres to the Medical Device Regulations (MDR) and has been released on the official app stores of Google (April 30, 2020, 8:25 p.m.) and Apple (April 24, 2020, 12:47 p.m.). The app collects anonymous information about users' sociodemographic characteristics, COVID-19–related symptoms, and recent close contact with an infected individual to provide direct feedback to users. This includes recommendations on how individuals should act, such as seeking medical advice, avoiding contact with others, and taking other protective measures. This study aims to describe the CoronaCheck user characteristics based on data from Germany, India, and South Africa and to determine associations of self-reports of COVID-19–related symptoms and previous contacts with individuals infected with COVID-19 with country, sociodemographic characteristics (gender identity, age group, educational level), calendar year, and user status (reported for oneself vs reported for another person).

## Methods

### Ethical Considerations

The study was approved by the Ethics Committee of the University of Würzburg (ethical approval no. 71/20-me) and the university’s data protection officer and was carried out in accordance with the General Data Protection Regulations of the European Union [23]. To have their anonymous data included in this study, all app users had to provide informed consent and agree that the data can be used for research purposes. With regard to data protection, users were also given comprehensive information at the beginning, and this information can also be viewed at any time [24].

### Experimental Design

CoronaCheck was developed in a scientific collaboration between 2 German university partners from Bavaria and software companies and is based on the openly available information about SARS-CoV-2 and corresponding recommendations of the Robert Koch Institute, the German national health authority [22]. CoronaCheck combines the ideas of patient-reported outcomes and mobile sensing with direct feedback from the app to users based on their answers to the questions. The overall goal is to provide a quick test that can be easily performed at any time and with any change in symptoms. In developing the app, it was important to comply with the MDR. The app was released in the official app stores of Google and Apple and complies with the MDR.

On April 8, 2020 (10 weeks after the first COVID-19 cases were reported in Germany), data collection from this app began in Germany, followed by the first data from South Africa and India on April 24 and 28, 2020, respectively.

### Ascertainment of Demographic Information

We collected data on age (years, categorized in 10-year intervals up to 79 years, ie, ≤9, 10-19, 20-29, 30-39, 40-49, 50-59, 60-69, 70-79, ≥80 years), gender identity (male, female, diverse, not specified), educational level (defined by years of schooling in 4 categories: ≤9 years, 10-11 years, ≥12 years, and “not reported” if users did not answer this question), and the information for whom the questionnaire was filled out (oneself, another person, not specified).

### Ascertainment of COVID-19–Associated Symptoms

Participants were asked about the presence of 11 different COVID-19–related symptoms in the past 24 hours (yes/no): fever (defined as a temperature of 37.5 °C or more), sore throat, stuffed or runny nose, cough, loss of smell, loss of taste, shortness of breath, headache, muscle or joint pain, diarrhea, and general weakness. In the case of diarrhea, the symptom was assessed only in the first 2 months (April-June 2020), before it was deleted in accordance with the recommendations of the Robert Koch Institute.

### Assessment of Close Contact With a Confirmed Case of COVID-19

We asked participants whether they or the person for whom they completed the questionnaire had had close contact with a person with confirmed COVID-19 in the past 14 days (yes, no, or not specified). We did not ask whether this confirmed case was based on a polymerase chain reaction (PCR) test, a medical diagnosis (including clinical radiology tests), or other information.

### App Development and Illustrations

**Figure 1:**
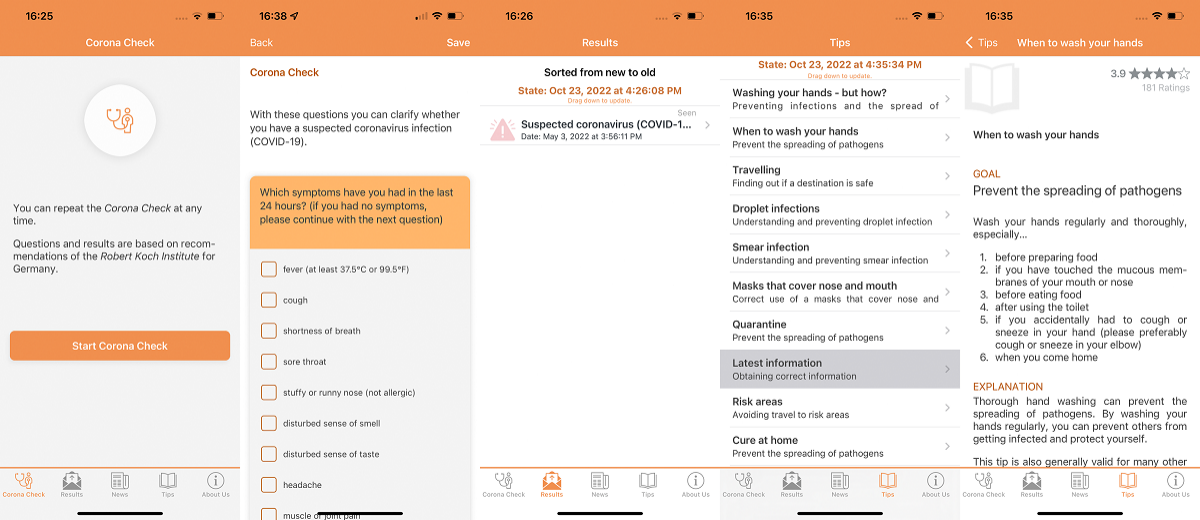
App illustration: screenshots of (1) Start Check, (2) Questionnaire, (3) Feedback Module, (4) Tips, and (5) Detailed Tip.

The app was developed based on the TrackYourHealth platform [20,21]. This always consists of a native iOS (Objective C) and a native Android (Java) app as well as a server backend. The latter includes a relational database and interface for mobile apps. The interface is based on the representational state transfer (REST) architectural style, and the connection to the apps is carried out via an encrypted secure sockets layer (SSL) channel [25]. The apps include a questionnaire for the COVID-19 self-check and a module that calculates the feedback for the symptoms. The module consists of the server-side calculation and a component in the apps that displays the feedback. Furthermore, 2 other modules are enabled in the app, one that displays general news located on the server and one that provides tips about COVID-19. The tips are intended to make everyday life easier and can also be evaluated by users in terms of their usefulness. Finally, the app provides information about the team, privacy, and declarations of conformity. The server for the data acquisition is located at the Service Center Medical Informatics at the University Hospital in Würzburg (SMI), and the app has been published in the official stores of Apple and Google. The last thing to mention is that all information (ie, the questionnaire, feedbacks, tips, news) is cached on the mobile devices, but server-side changes appear as an update in the app. Figure 1 shows screenshots of the app. The first screenshot shows the screen to start the test, the second the test in action, the third the feedback screen, the fourth the list of tips, and the fifth a detail page for a tip.

### Data Management

Only participants who met the following criteria were included in the final analysis: provided consent to use data for research purposes; answered questions about age, user status, and gender identity; provided GPS data; and answered questions about previous contacts (in the past 14 days) with a person infected with COVID-19 and the presence or absence of the 11 symptoms listed before. In case the app was used multiple times by a single person, only the first recording was used. It should be noted that every time a questionnaire is filled out, the GPS location is saved, provided that the user gives their consent. The location permission is regulated by the operating system (Android or iOS). We do not store the raw location, but we process the data we receive from the operating system to make it more coarse-grained, and only store a value with an accuracy of 11.1 km. The level of detail with which we store the GPS sensor data was determined in discussions with all stakeholders, the institutional review board, and relevant laws and regulations. Further, only data from countries with more than 3000 users (Germany, India, and South Africa) were included in the statistical analyses. Data from other countries were comparatively small (each <600 users). The educational level was categorized into 3 groups (<12 years, ≥12 years, missing). Since most reports were collected in 2020 and the first variants of concern emerged around the turn of 2020/2021 [26], we defined 2 categories (2020 and 2021/2022) for the calendar year variable.

### Statistical Analysis

Descriptive statistics were conducted to describe the CoronaCheck user characteristics. To compare sociodemographic characteristics (gender identity, age, education), calendar year, and user status among app users from Germany, India, South Africa, and other countries, chi-square tests were applied. Chi-square tests and Fisher exact tests were performed to compare the proportion of users who reported COVID-19–related symptoms and contact with a person infected with COVID-19 between 3 countries (Germany, India, and South Africa), gender identity (3 categories), educational level (3 categories), calendar years (2020 vs 2021/2022), and user status (symptoms reported for oneself vs another person). Univariate logistic regressions were calculated to assess the associations between reporting COVID-19–related symptoms and reporting contact with a person who tested positive for COVID-19 with country, gender, age (in 10-year categories), calendar year, educational level, and user status. Multivariable logistic regression was conducted to adjust the data for country, gender identity, age, calendar year, education level, and user status. Odds ratios (ORs) and their 95% CIs were estimated to assess the statistical uncertainty. All statistical analyses were performed using SPSS version 26 (IBM Corp), and all tests were 2-tailed.

## Results

### Study Population

From April 8, 2020, to February 3, 2022, we received a total of 88,537 recordings. Recordings with missing GPS data (n=29,449, 33.3%), missing consent that data can be used for research purposes (n=30,836, 34.8%), or missing information about age (n=2001, 2.3%), gender identity (n=1927, 2.2%), or user status (n=10,039, 11.3%) were excluded. Only the first recording per person was included in the case of multiple use. After applying these exclusion criteria, 23,179 recordings remained. All recordings included answers on the questions about previous contact (in the past 14 days) with a person infected with COVID-19 and the presence or absence of the 11 symptoms listed before. Most recordings were from Germany (n=8116, 35.0%), India (n=6622, 28.6%), and South Africa (n=3705, 16.0%). Recordings from 131 other countries ranged from 1 (0.004%) to 595 (2.6%) per country and were summarized into 1 group, “other countries” (n=4736, 20.4%).

The characteristics of the sample stratified by country are summarized in Table 1. In short, about half of the users were younger than 30 years old, with a higher proportion of young people using the app in India than in Germany. On average, 35.5% (n=8218) of the users were women, with the lowest proportion of women using the app in India.

The 4736 (20.4%) app users from “other countries” were excluded from further analysis, yielding a final analytic cohort of 18,443 (79.6%) individuals.

**Table 1:** Characteristics of CoronaCheck app users stratified by country.

Characteristics			Country									Total (N=23,179), n (%)
			Germany (n=8116, 35.0%), n (%)		India (n=6622, 28.6%), n (%)		South Africa (n=3705, 16.0%), n (%)		Other countries (n=4736, 20.4%), n (%)			
**Gender identity (*P*<.001)**												
	Man	4769 (58.8)		4686 (70.8)		2082 (56.2)		3305 (69.8)		14,842 (64.0)		
	Woman	3308 (40.8)		1893 (28.6)		1611 (43.5)		1406 (29.7)		8218 (35.5)		
	Diverse	39 (0.5)		43 (0.6)		12 (0.3)		25 (0.5)		119 (0.5)		
**Age (years; *P*<.001)**												
	< 30	1964 (24.2)		4649 (70.2)		1982 (53.5)		2507 (52.9)		11,102 (47.9)		
	30-49	2722 (33.5)		1556 (23.5)		1469 (39.6)		1480 (31.4)		7227 (31.2)		
	≥50	3430 (42.3)		417 (6.3)		254 (6.9)		749 (15.8)		4850 (20.9)		
**Education (years; *P*<.001)**												
	<12	3827 (47.2)		1849 (27.9)		891 (24.0)		1372 (29.0)		7939 (34.3)		
	≥12	3030 (37.3)		2047 (30.9)		1906 (51.4)		1970 (41.6)		8953 (38.6)		
	Missing	1259 (15.5)		2726 (41.2)		908 (24.5)		1394 (29.4)		6287 (27.1)		
**Calendar year (*P*<.001)**												
	2020	7084 (87.3)		5805 (87.7)		3094 (83.5)		3740 (79.0)		19,723 (85.1)		
	2021/2022	1032 (12.7)		817 (12.3)		611 (16.5)		996 (21.0)		3456 (14.9)		
**User status (*P*<.001)**												
	Reported for oneself	7499 (92.4)		5222 (78.9)		3314 (89.4)		4032 (85.1)		20,067 (86.6)		
	Reported for another person	617 (7.6)		1400 (21.1)		391 (10.6)		704 (14.9)		3112 (13.4)		

### Occurrence of COVID-19–Related Symptoms and Close Contact With an Infected Person

Of the 18,443 recordings collected in Germany, India, and South Africa, the 3 most frequent self-reported symptoms were headache (n=5351, 29.0%), cough (n=5219, 28.3%), and general weakness (n=4592, 24.9%), followed by muscle pain (n=4131, 22.4%), runny nose (n=3984, 21.6%), fever (n=3815, 20.7%), and sore throat (n=3646, 19.8%). Shortness of breath (n=2852, 15.5%), loss of smell (n=2224, 12.1%), loss of taste (n=2161, 11.7%), and diarrhea (n=136, 0.7%) were reported less frequently.

In 5105 (27.7%) of the 18,443 recordings collected in the 3 countries, the app users reported close contact with someone infected with SARS-CoV-2 in the past 14 days.

### Exploration of Country Differences

**Figure 2:**
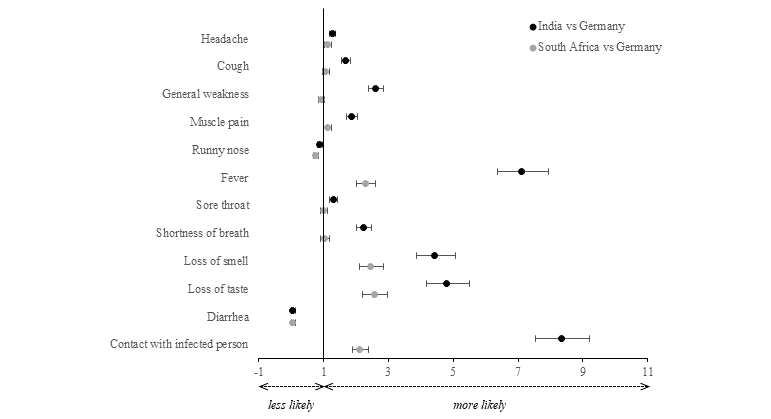
aORs and their 95% CIs stratified by the country of the CoronaCheck app users. aOR: adjusted odds ratio.

We found differences between the 3 countries in all symptoms (Figure 2 and Multimedia Appendix 1, Tables S1 and S2). We observed higher odds of reporting headache, cough, general weakness, muscle pain, fever, sore throat, shortness of breath, and loss of smell and taste: the adjusted odds ratios (aORs) were 1.27-7.10 (95% CI 1.17-7.95) for app users from India compared to Germany. In contrast, app users from India reported diarrhea less frequently than those from Germany (aOR 0.05, 95% CI 0.02-0.12). App users from South Africa were more likely to report headache, muscle pain, fever, and loss of smell and taste (aORs 1.11-2.56, 95% CI 1.00-2.98), while they were less likely to report runny nose (aOR 0.76, 95% CI 0.68-0.84) and diarrhea (aOR 0.03, 95% CI 0.01-0.12) than users from Germany. App users from India were significantly more likely to have knowledge of close contact with a person positive for SARS-CoV-2 in the past 14 days compared to Germany (aOR 8.33, 95% CI 7.53-9.21). To a lesser extent, this was also the case for app users from South Africa compared to Germany (aOR 2.12, 95% CI 1.89-2.38).

### Exploration of Sociodemographic Differences

#### Gender Identity Differences

**Figure 3:**
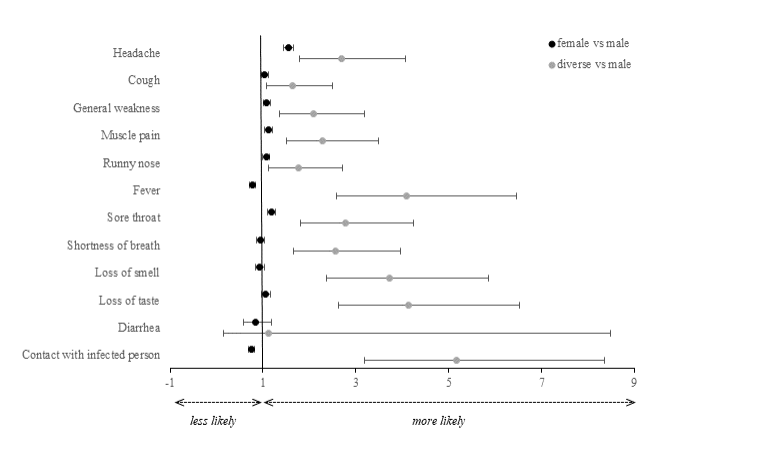
aORs and their 95% CIs stratified by the gender identity of the CoronaCheck app users. aOR: adjusted odds ratio.

Our results showed gender identity differences for all symptoms except diarrhea (Figure 3 and Multimedia Appendix 1, Tables S3 and S4). Women app users reported fever less often (aOR 0.78, 95% CI 0.72-0.85) and headache, general weakness, muscle pain, runny nose, and sore throat more often (aORs 1.07-1.55, 95% CI 1.00-1.65) compared to men. Except for diarrhea, gender-diverse users reported COVID-19–related symptoms more often compared to men (aORs 1.64-4.14, 95% CI 1.08-6.53). Women were less likely than men to know of recent contact with a person infected with SARS-CoV-2 (aOR 0.76, 95% CI 0.70-0.82). Gender-diverse users had 5.16 times higher odds (95% CI 3.18-8.36) of reporting close contact with a person infected with SARS-CoV-2 in the past 14 days compared to app users who were men.

#### Age Effects

Older persons were less likely to report headache, cough, general weakness, runny nose, fever, sore throat, and loss of smell (aORs 0.86-0.96, 95% CI 0.84-0.99, per 10-year category), whereas they were more likely to report muscle pain (aOR 1.03, 95% CI 1.01-1.05, per 10-year category; Table 2). Older persons were less likely to report close contact with a person positive for SARS-CoV-2 in the past 14 days (aOR 0.87, 95% CI 0.85-0.89, per 10-year category).

**Table 2:** Unadjusted ORs^a^ and aORs^b^ by 10-year age category (N=18,445).^c^

Variable	Unadjusted OR (95% CI)	aOR (95% CI)
Headache	0.84 (0.82-0.86)	0.87 (0.85-0.89)
Cough	0.85 (0.84-0.87)	0.90 (0.89-0.92)
General weakness	0.87 (0.86-0.89)	0.96 (0.94-0.99)
Muscle pain	0.96 (0.94-0.98)	1.03 (1.01-1.05)
Runny nose	0.87 (0.85-0.89)	0.86 (0.84-0.88)
Fever	0.76 (0.74-0.77)	0.92 (0.90-0.94)
Sore throat	0.84 (0.82-0.86)	0.87 (0.85-0.89)
Shortness of breath	0.91 (0.89-0.93)	0.99 (0.97-1.02)
Loss of smell	0.81 (0.79-0.83)	0.95 (0.92-0.98)
Loss of taste	0.83 (0.81-0.86)	0.98 (0.95-1.02)
Diarrhea	1.17 (1.08-1.28)	0.92 (0.84-1.01)
Contact with infected person	0.71 (0.70-0.73)	0.87 (0.85-0.89)

^a^OR: odds ratio.

^b^aOR: adjusted odds ratio.

^c^The multivariable regression model was adjusted for country, gender identity, calendar year, education, and user status.

#### Differences Concerning the Educational Level

**Figure 4:**
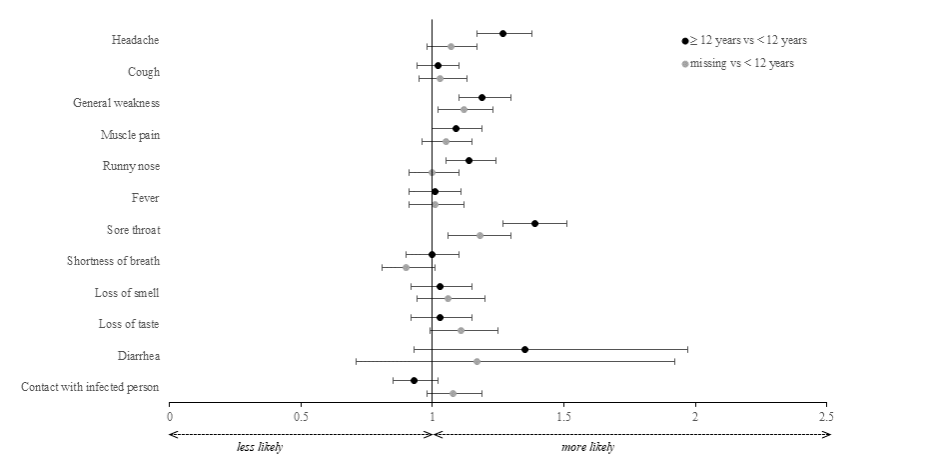
aORs and their 95% CIs stratified by educational level (years of schooling). aOR: adjusted odds ratio.

App users with at least 12 years of school education reported headache, general weakness, muscle pain, runny nose, and sore throat more often compared to users with 11 or fewer years of education (aORs 1.09-1.39, 95% CI 1.00-1.51; Figure 4 and Multimedia Appendix 1, Tables S5 and S6).

### Exploration of Differences Between Calendar Years

**Figure 5:**
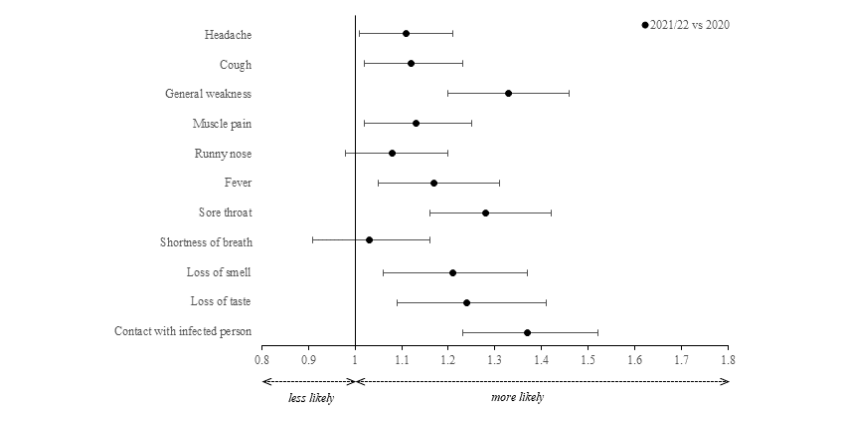
aORs and their 95% CIs stratified by the year of using the CoronaCheck app. aOR: adjusted odds ratio.

Most COVID-19–associated symptoms were more likely to be reported by app users in 2021/2022 compared to 2020 (aORs 1.11-1.33, 95% CI 1.01-1.46; Figure 5 and Multimedia Appendix 1, Tables S7 and S8). The occurrence of runny nose and shortness of breath was not associated with the calendar year.

The reporting of close contact with someone infected with SARS-CoV-2 in the past 14 days was more likely in 2021/2022 compared to 2020 (aOR 1.37, 95% CI 1.23-1.52).

### Exploration of Differences Concerning the Status of the Person (Reported for Oneself vs for Others)

**Figure 6:**
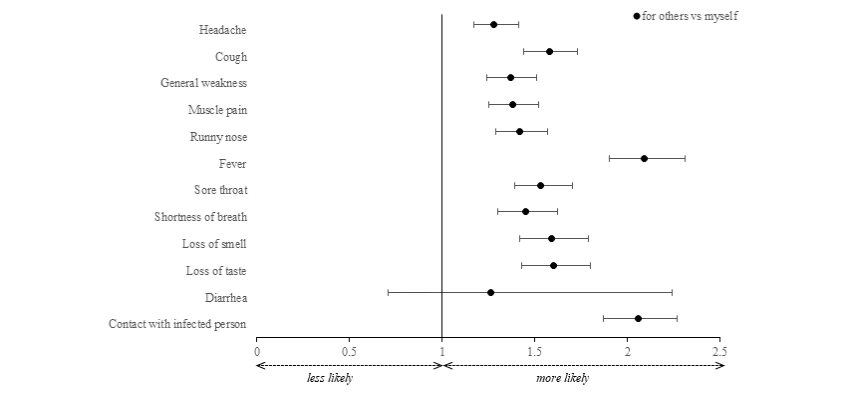
aORs and their 95% CIs stratified by user status (ie, user-reported symptoms for another person or for oneself). aOR: adjusted odds ratio.

We found differences between persons for whom symptoms were reported for all symptoms except diarrhea (Figure 6 and Multimedia Appendix 1, Tables S9 and S10).

Symptoms as well as close contact with a person positive for SARS-CoV-2 in the past 14 days were more often reported when the data were entered for another person compared to for oneself (aORs 1.28-2.09, 95% CI 1.17-2.31).

## Discussion

### Principal Findings

Our study showed that self-reported COVID-19–related symptoms and reports of previous contact with individuals infected with COVID-19 vary by country, gender identity, age, calendar year, education, and user status.

Overall, the most frequently reported symptoms were headache, cough, and general weakness. Symptoms commonly included in COVID-19 case definitions, such as shortness of breath and fever [27,28], were less frequently reported by app users. Especially app users from India (compared to Germany), as well as gender-diverse (compared to men) users were more likely to report COVID-19–related symptoms and previous close contact with infected individuals.

### Comparison With Prior Work

Our findings regarding the occurrence of common symptoms, such as headache, general weakness, and runny nose, are consistent with previous studies. Specifically, previous studies in individuals infected with PCR-confirmed SARS-CoV-2 have shown that fatigue, headache, malaise, myalgia, and upper respiratory symptoms (sore throat, cough, sneezing, rhinitis) occur early after symptom onset, whereas symptoms considered more characteristic of COVID-19, such as lower respiratory and chemosensory symptoms, occur later [17,29,30].

Our study showed differences in self-reported COVID-19 symptoms primarily between Germany, India, and South Africa. App use from India and South Africa was associated with a disproportionately high likelihood of experiencing COVID-19–related symptoms and knowledge of close contact with an infected person. Specifically, the likelihood of reporting contact with a person infected with COVID-19 was 8.3-fold higher among users from India compared to Germany. Most symptoms were also more likely to be reported by users from India than by German users. World Health Organization (WHO) official data on confirmed COVID-19 cases show conflicting results, with higher numbers of confirmed cases and deaths per capita in Germany than in India and South Africa [2]. In more detail, by the beginning of February 2022, the cumulative confirmed COVID-19 cases amounted to 15% of the German population, higher than in South Africa (6%) and India (3%). The discrepancy is likely due to several factors of uncertainty in the COVID-19 data, such as the low number of COVID-19 tests and deficiencies in surveillance systems in India that result in underreporting of infections and deaths [31]. Studies that compare cross-national cases run the risk of reaching incorrect conclusions [19]. Thus, a higher likelihood of reporting a previous contact with a person testing positive for COVID-19 in our study and the reporting of symptoms associated with COVID-19 suggest that there is substantial underreporting bias in the existing data from India. In addition, higher odds of reporting previous contact with a person positive for COVID-19 (OR 2.12) as well as higher odds of reporting several symptoms were observed for app users from South Africa compared to Germany. Although South Africa is the country that performs the most COVID-19 testing among African countries and is one of the few countries that produces vital statistics, it is likely that the number of COVID-19 cases and deaths is underestimated [32]. The findings suggest that effective management of the pandemic is particularly difficult in countries with limited resources, inadequate testing, and weak health systems.

Gender identity differences were found in this study. Several symptoms were more likely to be reported by women and gender-diverse users than by men. Exceptions to the general assumption that women are more likely to report symptoms than men were observed for fever. Previous studies of individuals testing positive for COVID-19 support the assumption that women are more likely than men to be affected by headache and sore throat, as well as rhinitis [15,17,33,34]. Results regarding muscle pain [15,17] are equivocal, and fever seems to be less prevalent in women [33]. Female users less frequently reported contact with a person positive for COVID-19 compared to male users. Previous studies highlight that women are more willing to adhere to protective measures [35,36], which might be 1 explanation for the lower odds of self-reported contact with an infected person in women individuals and possibly also to the overall lower proportion of women app users.

The impact of COVID-19 on COVID-19 symptoms in gender-diverse individuals has not been well captured; however, the more than 5-fold increase in the likelihood of gender-diverse users reporting prior contact with an individual who tested positive for COVID-19, as well as the higher odds of reporting symptoms observed in this study, is supported by the notion that gender-diverse individuals are more vulnerable to COVID-19, which extends to the areas of increased barriers to COVID-19 transmission prevention strategies, decreased physical and mental health, and barriers to health services [37]. As such, the higher odds of reporting contact with persons infected with COVID-19 corroborates the reported reduced options of gender-diverse individuals to practice physical distancing, as they are more likely to be essential workers, requiring in-person work and often commuting by public transport [37]. Other discussed aspects are the higher behavioral risks, such as substance use and smoking, which are common among gender-diverse people [38]. Studies have also reported a higher risk for chronic health conditions, such as diabetes, in gender-diverse individuals [39], and they often face barriers to health care services [40]. Greater vaccination hesitancy has also been reported, likely associated with medical mistrust due to the historical mistreatment of gender-diverse individuals [37]. Thus, all these factors might place this population at greater risk for COVID-19–related illness. Considering these previous reports as well as the findings of the study at hand, gender-diverse individuals seem to be among those minorities most strongly affected by the COVID-19 pandemic.

Older age was found to be associated with a lower likelihood of reporting contact with a person infected with COVID-19. In support of this finding, previous studies have found an association between older age and self-reported adherence to COVID-19 public health measures [41]). Reports of age-related differences in the clinical presentation of patients with COVID-19 suggest that older patients with COVID-19 are more likely to have general symptoms (fever, fatigue, gastrointestinal symptoms) than specific symptoms (loss of smell and taste) [17,33,34]. In this study, the odds of reporting almost all COVID-19–related symptoms decreased with increasing age. Given the lower likelihood of being a contact in older individuals, it can be surmised that the proportion of app users infected with COVID-19 was lower among older users, which likely contributed to their lower frequency of reported symptoms.

The increased odds of reporting contact with someone who tested positive for SARS-CoV-2 in 2021/2022 compared to 2020 is supported by the increased transmission of the variants of concern, which predominated from 2021 onward. The Alpha variant (B.1.1.7), first identified in the United Kingdom at the end of 2020; the Beta variant (B.1.351), first identified in South Africa at the end of 2020; the Delta variant (B.1.617.2), first identified in India at the end of 2020; and the Omicron variant (B.1.1.529), first identified in South Africa at the end of 2021, have roughly 50% increased transmission [11]. In addition, the higher odds of reporting symptoms after 2020 compared to the year in which the SARS-CoV-2 wild-type virus was dominant might be due to the increased disease severity of the Alpha, Beta, and Delta variants [26].

In this study, the person for whom the symptom check was used affected the outcome: the likelihood of reporting contact with a person positive for COVID-19 and various COVID-19–related symptoms was higher when the app was used to check symptoms for another person. Thus, it appears that the app was used more frequently for other individuals when there was a specific suspicion of infection. Differences in reporting systems have been observed previously, with a higher percentage of symptoms reported in self-reports than in physician reports [7]. Therefore, the use of a self-report app seems to be an appropriate method for recording COVID-19–related symptoms.

### Strengths

Our study had several strengths. First, data were collected over a long time interval that began early in the pandemic. Data were collected across different countries, with considerable differences in health care systems, economic status, and governmental regulations to combat the spread of the virus, therefore increasing the generalizability of the findings. The number of gender-diverse individuals among app users also allowed us to identify the specifics of symptoms reported by COVID-19 among gender minorities. In addition, it was possible to develop the app in a relatively short period and adapt it to the MDR, which meant that, among other things, special consideration was given to the risks involved in using the app. The app made use of GPS measurements when users allowed it, which shows that such measurements are also welcomed by many users. Finally, the content of the app was based on the recommendations of the Robert Koch Institute, the national public health institute in Germany. We therefore regularly adapted the app to the changing official recommendations.

### Limitations

Our study also had some limitations that need to be noted. First, objective data on whether app users tested positive for COVID-19 or not were not available. Thus, we were not able to validate the subjectively reported information about COVID-19 test positivity. Moreover, no objective data were available if the infected contact person of the user had a positive COVID-19 test; however, the corresponding question asked specifically about contact with a person who was a confirmed case.

Second, all information about specific symptoms was self-reported by the users, which was not validated by, for example, doctors’ diagnoses. In those cases where the questionnaire was filled out for another person, the validity of the data is further limited. This especially applied to the youngest and oldest app users, where a higher proportion of reported information was not entered by themselves (51.5% among children ≤9 years old and 21.1% among the elderly ≥80 years old vs 12.1% among 10-79-year-old persons).

Third, participants’ socioeconomic status was limited to the information about the years of education in school. This information was further limited by the high number of missing entries as well as by differences in the educational systems between countries.

Fourth, we had to adjust the app several times to meet the changing official recommendations of the German public health authority, the Robert Koch Institute, over the course of the pandemic. Therefore, during the overall time of app usage, especially in the early phase of the pandemic, some information was slightly differently presented to users. For example, the question regarding diarrhea was only administered for the first 2 months before it was removed.

Furthermore, as data collection was based on a mobile app, results might not be generalized to individuals with lower affinity for digital health apps. In concrete terms, this also means that information and selection bias must be taken into account. However, observer and recall bias can be reduced to some extent by an app in terms of patient-reported outcomes. Therefore, app-based data have strengths as well as weaknesses that need to be considered. Moreover, mHealth apps are always not just tools but also social phenomena that create a feedback loop between app and user, which means that users continuously adjust their behavior as they use the app. However, since the CoronaCheck app has collected a comparatively large amount of data in different cultures compared to other mHealth apps, certain differences may have been balanced out in the sense of generalization. Finally, it should be mentioned that apps that are made available on multiple mobile operating systems will also always generate differences in the resulting data, as the user interface is never completely identical.

### Conclusion

Our app-based results indicated country-specific differences in COVID-19–related symptom patterns. It seems that self-reported COVID-19–related symptoms and awareness about previous contacts with individuals infected with COVID-19 are more likely among app users from India and South Africa (compared to users from Germany), as well as in gender-diverse (compared to male) users. Symptom assessment tools could play a positive role in the context of widespread infectious diseases. In general, with COVID-19, we saw that the way symptoms were reported and the way infection and COVID-19 deaths were defined were different [42]. For future pandemics, app-based global data collection, such as that of CoronaCheck, is another tool to better equip us and support appropriate assessments.

Future mHealth research on country-specific differences during a viral pandemic should aim to recruit representative samples. If pseudonymous assessments are possible, studies should collect information about symptoms, confirmed testing, and adherence to public health recommendations longitudinally.

## Appendix

Multimedia Appendix 1 Supplementary tables.

## Abbreviations

aOR: adjusted odds ratio
MDR: Medical Device Regulations
mHealth: mobile health
OR: odds ratio
PCR: polymerase chain reaction

## Data Availability

The data presented in this study are only available on request from the corresponding author. The data are not publicly available, because participants’ informed consent did not cover public deposition of data.

## References

[1] Li J, Lai S, Gao GF, Shi W (2021). The emergence, genomic diversity and global spread of SARS-CoV-2. Nature.

[2] World Health Organization WHO Coronavirus (COVID-19) Dashboard.

[3] John Leon Singh H, Couch D, Yap K (2020). Mobile health apps that help with COVID-19 management: scoping review. JMIR Nurs.

[4] Mary L, Raj S (2021). A survey on SARS-CoV-2 (COVID-19) using machine learning techniques.

[5] Spinato G, Gaudioso P, Boscolo Rizzo P, Fabbris C, Menegaldo A, Mularoni F, Singh B, Maniaci A, Cocuzza S, Frezza D (2021). Risk management during COVID-19: safety procedures for otolaryngologists. Acta Biomed.

[6] Grant MC, Geoghegan L, Arbyn M, Mohammed Z, McGuinness L, Clarke EL, Wade RG (2020). The prevalence of symptoms in 24,410 adults infected by the novel coronavirus (SARS-CoV-2; COVID-19): a systematic review and meta-analysis of 148 studies from 9 countries. PLoS One.

[7] Mizrahi B, Shilo S, Rossman H, Kalkstein N, Marcus K, Barer Y, Keshet A, Shamir-Stein N, Shalev V, Zohar AE, Chodick G, Segal E (2020). Longitudinal symptom dynamics of COVID-19 infection. Nat Commun.

[8] Callejon-Leblic MA, Moreno-Luna R, Del Cuvillo A, Reyes-Tejero IM, Garcia-Villaran MA, Santos-Peña M, Maza-Solano JM, Martín-Jimenez DI, Palacios-Garcia JM, Fernandez-Velez C, Gonzalez-Garcia J, Sanchez-Calvo JM, Solanellas-Soler J, Sanchez-Gomez S (2021). Loss of smell and taste can accurately predict COVID-19 infection: a machine-learning approach. J Clin Med.

[9] Sudre CH, Keshet A, Graham MS, Joshi AD, Shilo S, Rossman H, Murray B, Molten E, Klaser K, Canas LD, Antonelli M, Nguyen LH, Drew DA, Modat M, Pujol JC, Ganesh S, Wolf J, Meir T, Chan AT, Steves CJ, Spector TD, Brownstein JS, Segal E, Ourselin S, Astley CM (2021). Anosmia, ageusia, and other COVID-19-like symptoms in association with a positive SARS-CoV-2 test, across six national digital surveillance platforms: an observational study. Lancet Digital Health.

[10] Elias C, Sekri A, Leblanc P, Cucherat M, Vanhems P (2021). The incubation period of COVID-19: a meta-analysis. Int J Infect Dis.

[11] Long B, Carius BM, Chavez S, Liang SY, Brady WJ, Koyfman A, Gottlieb M (2022). Clinical update on COVID-19 for the emergency clinician: presentation and evaluation. Am J Emerg Med.

[12] Quer G, Radin JM, Gadaleta M, Baca-Motes K, Ariniello L, Ramos E, Kheterpal V, Topol EJ, Steinhubl SR (2021). Wearable sensor data and self-reported symptoms for COVID-19 detection. Nat Med.

[13] Rai P, Kumar BK, Deekshit VK, Karunasagar I, Karunasagar I (2021). Detection technologies and recent developments in the diagnosis of COVID-19 infection. Appl Microbiol Biotechnol.

[14] Almalki M, Giannicchi A (2021). Health apps for combating COVID-19: descriptive review and taxonomy. JMIR Mhealth Uhealth.

[15] Sebo P, Maisonneuve H, Lourdaux J, Cuzin C, Floquet M, Tudrej B, Haller D (2021). Self-reported symptoms in French primary care SARS-CoV-2 patients: association with gender and age group. Fam Pract.

[16] Ahmed MM, Sayed AM, El Abd D, Fares S, Said MSM, Elsayed Sedik Ebrahim E (2021). Gender difference in perceived symptoms and laboratory investigations in suspected and confirmed COVID-19 cases: a retrospective study. J Prim Care Community Health.

[17] Wiegele PN, Kabar I, Kerschke L, Froemmel C, Hüsing-Kabar A, Schmidt H, Vorona E, Vollenberg R, Tepasse P (2021). Symptom diary-based analysis of disease course among patients with mild coronavirus disease, Germany, 2020. Emerg Infect Dis.

[18] Buyukkececi Z (2020). Cross-country differences in anxiety and behavioral response to the Covid-19 pandemic. Eur Soc.

[19] Biswas RK, Afiaz A, Huq S (2020). Underreporting COVID-19: the curious case of the Indian subcontinent. Epidemiol Infect.

[20] Vogel C, Pryss R, Schobel J, Schlee W, Beierle F (2021). Developing apps for researching the COVID-19 pandemic with the TrackYourHealth platform.

[21] Beierle F, Schobel J, Vogel C, Allgaier J, Mulansky L, Haug F, Haug J, Schlee W, Holfelder M, Stach M, Schickler M, Baumeister H, Cohrdes C, Deckert J, Deserno L, Edler J, Eichner FA, Greger H, Hein G, Heuschmann P, John D, Kestler HA, Krefting D, Langguth B, Meybohm P, Probst T, Reichert M, Romanos M, Störk S, Terhorst Y, Weiß M, Pryss R (2021). Corona Health—a study- and sensor-based mobile app platform exploring aspects of the COVID-19 pandemic. IJERPH.

[22] Holfelder M, Mulansky L, Schlee W, Baumeister H, Schobel J, Greger H, Hoff A, Pryss R (2021). Medical device regulation efforts for mhealth apps during the COVID-19 pandemic—an experience report of Corona Check and Corona Health. J.

[23] European Union (2016). Regulation (EU) 2016/679 of the European Parliament and of the Council of 27 April 2016 on the protection of natural persons with regard to the processing of personal data and on the free movement of such data, and repealing Directive 95/46/EC (General Data Protection Regulation). OJEU.

[24] Corona Health Privacy Policy.

[25] Pryss R, Schobel J, Reichert M (2018). Requirements for a flexible and generic API enabling mobile crowdsensing mHealth applications.

[26] Tao K, Tzou PL, Nouhin J, Gupta RK, de Oliveira T, Kosakovsky Pond SL, Fera D, Shafer RW (2021). The biological and clinical significance of emerging SARS-CoV-2 variants. Nat Rev Genet.

[27] Guan W, Ni Z, Hu Y, Liang W, Ou C, He J, Liu L, Shan H, Lei C, Hui DS, Du B, Li L, Zeng G, Yuen K, Chen R, Tang C, Wang T, Chen P, Xiang J, Li S, Wang J, Liang Z, Peng Y, Wei L, Liu Y, Hu Y, Peng P, Wang J, Liu J, Chen Z, Li G, Zheng Z, Qiu S, Luo J, Ye C, Zhu S, Zhong N, China Medical Treatment Expert Group for Covid-19 (2020). Clinical characteristics of coronavirus disease 2019 in China. N Engl J Med.

[28] Shen Y, Zheng F, Sun D, Ling Y, Chen J, Li F, Li T, Qian Z, Zhang Y, Xu Q, Liu L, Huang Q, Shan F, Xu L, Wu J, Zhu Z, Song Z, Li S, Shi Y, Zhang J, Wu X, Mendelsohn JB, Zhu T, Lu H (2020). Epidemiology and clinical course of COVID-19 in Shanghai, China. Emerg Microbes Infect.

[29] Saad N, Moek F, Steitz F, Murajda L, Bärnighausen T, Zoller T, Pörtner K, Muller N (2021). A longitudinal study on symptom duration and 60-day clinical course in non-hospitalised COVID-19 cases in Berlin, Germany, March to May, 2020. Eurosurveillance.

[30] Bliddal S, Banasik K, Pedersen OB, Nissen J, Cantwell L, Schwinn M, Tulstrup M, Westergaard D, Ullum H, Brunak S, Tommerup N, Feenstra B, Geller F, Ostrowski SR, Grønbæk K, Nielsen CH, Nielsen SD, Feldt-Rasmussen U (2021). Acute and persistent symptoms in non-hospitalized PCR-confirmed COVID-19 patients. Sci Rep.

[31] Chatterjee P (2020). Is India missing COVID-19 deaths?. Lancet.

[32] Okonji EF, Okonji OC, Mukumbang FC, Van Wyk B (2021). Understanding varying COVID-19 mortality rates reported in Africa compared to Europe, Americas and Asia. Trop Med Int Health.

[33] Lechien JR, Chiesa-Estomba CM, Place S, Van Laethem Y, Cabaraux P, Mat Q, Huet K, Plzak J, Horoi M, Hans S, Rosaria Barillari M, Cammaroto G, Fakhry N, Martiny D, Ayad T, Jouffe L, Hopkins C, Saussez S, COVID-19 Task Force of YO-IFOS (2020). Clinical and epidemiological characteristics of 1420 European patients with mild-to-moderate coronavirus disease 2019. J Intern Med.

[34] Lee Y, Min P, Lee S, Kim S (2020). Prevalence and duration of acute loss of smell or taste in COVID-19 patients. J Korean Med Sci.

[35] Coroiu A, Moran C, Campbell T, Geller AC (2020). Barriers and facilitators of adherence to social distancing recommendations during COVID-19 among a large international sample of adults. PLoS One.

[36] Lin T, Harris EA, Heemskerk A, Van Bavel JJ, Ebner NC (2021). A multi-national test on self-reported compliance with COVID-19 public health measures: the role of individual age and gender demographics and countries' developmental status. Soc Sci Med.

[37] Zubizarreta D, Trinh M, Reisner SL (2022). COVID-19 risk and resilience among U.S. transgender and gender diverse populations. Am J Prev Med.

[38] Buchting FO, Emory KT, Kim Y, Fagan P, Vera LE, Emery S, Scout (2017). Transgender use of cigarettes, cigars, and e-cigarettes in a national study. Am J Prev Med.

[39] Dragon CN, Guerino P, Ewald E, Laffan AM (2017). Transgender Medicare beneficiaries and chronic conditions: exploring fee-for-service claims data. LGBT Health.

[40] Jones BA, Bowe M, McNamara N, Guerin E, Carter T (2021). Exploring the mental health experiences of young trans and gender diverse people during the Covid-19 pandemic. Int J Transgend Health.

[41] Wright L, Fancourt D (2021). Do predictors of adherence to pandemic guidelines change over time? A panel study of 22,000 UK adults during the COVID-19 pandemic. Prev Med.

[42] HSC Public Health Agency Why Are There Differences in the Reporting of COVID-19 Related Deaths?.

