# Supplementary material for: Associations of Country-Specific and Sociodemographic Factors With Self-Reported COVID-19–Related Symptoms: Multivariable Analysis of Data From the CoronaCheck Mobile Health Platform
Source: JMIR Public Health Surveill. 2023 Feb 3;9:e40958. doi: 10.2196/40958 (PMC9901499; doi:10.2196/40958)
Supplement: Multimedia Appendix 1 [file publichealth_v9i1e40958_app1.docx]

Supplementary Table 1. Proportion of reported COVID-19 related symptoms and contacts with an infected person stratified by country (n = 18,443)

|  |  |  | Country | |  | |  | |
| --- | --- | --- | --- | --- | --- | --- | --- | --- |
| Variable |  | Germany  (n = 8,116) | India  (n = 6,622) | | South Africa  (n = 3,705) | | *P* | |
| Headache | % (n) | 24.3% (1,969) | | 33.7% (2,232) | | 31.0% (1,150) | | <.001 |
| Cough | % (n) | 21.9% (1,779) | | 37.3% (2,473) | | 26.0% (964) | | .017 |
| General weakness | % (n) | 17.4% (1,413) | | 38.0% (2,519) | | 17.8% (661) | | <.001 |
| Muscle pain | % (n) | 18.2% (1,478) | | 29.0% (1,920) | | 19.6% (727) | | <.001 |
| Runny nose | % (n) | 20.7% (1,676) | | 23.6% (1,562) | | 19.9% (738) | | <.001 |
| Fever | % (n) | 6.9% (560) | | 40.2% (2,662) | | 16.0% (593) | | <.001 |
| Sore throat | % (n) | 15.9% (1,289) | | 24.5% (1,624) | | 19.8% (733) | | <.001 |
| Shortness of breath | % (n) | 11.2% (907) | | 22.8% (1,512) | | 11.7% (433) | | <.001 |
| Loss of smell | % (n) | 4.8% (389) | | 21.1% (1,394) | | 11.9% (441) | | <.001 |
| Loss of taste | % (n) | 4.6% (374) | | 20.5% (1,360) | | 11.5% (427) | | <.001 |
| Diarrhea | % (n) | 1.6% (127) | | 0.1% (7) | | 0.1% (2) | | <.001 |
| Contact with infected person | % (n) | 9.3% (752) | | 54.2% (3,590) | | 20.6% (763) | | <.001 |

Supplementary Table 2. Unadjusted and adjusted odds ratios (ORs) stratified by country

|  | Unadjusted ORs | | | | | | Adjusted ORs | | | | | | | | | |
| --- | --- | --- | --- | --- | --- | --- | --- | --- | --- | --- | --- | --- | --- | --- | --- | --- |
| Variable | India | | | South Africa | | | India | | | South Africa | | | |  |  |  |
|  | OR | 95% CI | | OR | 95% CI | | OR | 95% CI | | OR | 95% CI | | | |  |  |
| Headache | 1.59 | 1.48 | 1.71 | 1.41 | 1.29 | 1.53 | 1.27 | 1.17 | 1.38 | 1.12 | | 1.02 | 1.23 | | |  |
| Cough | 2.12 | 1.98 | 2.28 | 1.25 | 1.15 | 1.37 | 1.68 | 1.54 | 1.83 | 1.07 | | 0.97 | 1.18 | | |  |
| General weakness | 2.91 | 2.70 | 3.14 | 1.03 | 0.93 | 1.14 | 2.61 | 2.39 | 2.86 | 0.93 | | 0.83 | 1.03 | | |  |
| Muscle pain | 1.83 | 1.70 | 1.98 | 1.10 | 0.99 | 1.21 | 1.86 | 1.70 | 2.04 | 1.11 | | 1.00 | 1.23 | | |  |
| Runny nose | 1.19 | 1.10 | 1.28 | 0.96 | 0.87 | 1.05 | 0.88 | 0.81 | 0.97 | 0.76 | | 0.68 | 0.84 | | |  |
| Fever | 9.07 | 8.22 | 10.01 | 2.57 | 2.27 | 2.91 | 7.10 | 6.35 | 7.95 | 2.28 | | 2.01 | 2.60 | | |  |
| Sore throat | 1.72 | 1.59 | 1.87 | 1.31 | 1.18 | 1.44 | 1.29 | 1.17 | 1.42 | 1.00 | | 0.90 | 1.12 | | |  |
| Shortness of breath | 2.35 | 2.15 | 2.57 | 1.05 | 0.93 | 1.19 | 2.24 | 2.01 | 2.49 | 1.04 | | 0.91 | 1.18 | | |  |
| Loss of smell | 5.30 | 4.71 | 5.96 | 2.68 | 2.33 | 3.09 | 4.43 | 3.87 | 5.07 | 2.44 | | 2.10 | 2.84 | | |  |
| Loss of taste | 5.35 | 4.75 | 6.03 | 2.70 | 2.33 | 3.12 | 4.80 | 4.18 | 5.50 | 2.56 | | 2.19 | 2.98 | | |  |
| Diarrhea | 0.07 | 0.03 | 0.14 | 0.03 | 0.01 | 0.14 | 0.05 | 0.02 | 0.12 | 0.03 | | 0.01 | 0.12 | | |  |
| Contact with infected person | 11.60 | 10.61 | 12.68 | 2.54 | 2.28 | 2.83 | 8.33 | 7.53 | 9.21 | 2.12 | | 1.89 | 2.38 | | |  |

Note: The multivariable regression model was adjusted for gender, age, year, education, and user status with Germany as reference group.

Supplementary Table 3. Proportion of reported COVID-19 related symptoms and contacts with an infected person stratified by gender (n = 18,443)

|  | |  | | | Gender | | | | |  | |  |
| --- | --- | --- | --- | --- | --- | --- | --- | --- | --- | --- | --- | --- |
| Variable | |  | Male  (n = 11,537) | | | Female  (n = 6,812) | | Diverse  (n = 94) | | *P* | |  |
| Headache | % (n) | | | 25.6% (2,953) | | | 34.5% (2,352) | | 48.9% (46) | | <.001 | |
| Cough | % (n) | | | 28.2% (3,257) | | | 28.2% (1,919) | | 42.6% (40) | | .009 | |
| General weakness | % (n) | | | 25.1% (2,899) | | | 24.3% (1,653) | | 43.6% (41) | | <.001 | |
| Muscle pain | % (n) | | | 22.1% (2,546) | | | 22.6% (1,539) | | 42.6% (40) | | <.001 | |
| Runny nose | % (n) | | | 20.9% (2,406) | | | 22.6% (1,538) | | 34.0% (32) | | <.001 | |
| Fever | % (n) | | | 22.8% (2,636) | | | 16.6% (1,128) | | 54.3% (51) | | <.001 | |
| Sore throat | % (n) | | | 18.7% (2,158) | | | 21.3% (1,449) | | 41.5% (39) | | <.001 | |
| Shortness of breath | % (n) | | | 16.0% (1,851) | | | 14.2% (967) | | 36.2% (34) | | <.001 | |
| Loss of smell | % (n) | | | 12.7% (1,464) | | | 10.7% (726) | | 36.2% (34) | | <.001 | |
| Loss of taste | % (n) | | | 11.9% (1,375) | | | 11.0% (751) | | 37.2% (35) | | <.001 | |
| Diarrhea | % (n) | | | 0.7% (85) | | | 0.7% (50) | | 1.1% (1) | | .933 | |
| Contact with infected person | % (n) | | | 30.6% (3,528) | | | 22.2% (1,515) | | 66.0% (62) | | <.001 | |

Supplementary Table 4. Unadjusted and adjusted odds ratios (ORs) stratified by gender

|  | Unadjusted ORs | | | | | | Adjusted ORs | | | | | |
| --- | --- | --- | --- | --- | --- | --- | --- | --- | --- | --- | --- | --- |
|  | female | | | diverse | | | female | | | diverse | | |
| Variable | OR | 95% CI | | OR | 95% CI | | OR | 95% CI | | OR | 95% CI | |
| Headache | 1.53 | 1.44 | 1.64 | 2.79 | 1.86 | 4.18 | 1.55 | 1.45 | 1.65 | 2.69 | 1.78 | 4.08 |
| Cough | 1.00 | 0.93 | 1.07 | 1.88 | 1.25 | 2.84 | 1.04 | 0.97 | 1.11 | 1.64 | 1.08 | 2.50 |
| General weakness | 0.96 | 0.89 | 1.02 | 2.31 | 1.53 | 3.47 | 1.08 | 1.01 | 1.17 | 2.08 | 1.36 | 3.19 |
| Muscle pain | 1.03 | 0.96 | 1.11 | 2.62 | 1.73 | 3.95 | 1.11 | 1.03 | 1.20 | 2.28 | 1.50 | 3.48 |
| Runny nose | 1.11 | 1.03 | 1.19 | 1.96 | 1.28 | 3.01 | 1.07 | 1.00 | 1.15 | 1.76 | 1.13 | 2.72 |
| Fever | 0.67 | 0.62 | 0.72 | 4.01 | 2.66 | 6.02 | 0.78 | 0.72 | 0.85 | 4.09 | 2.58 | 6.47 |
| Sore throat | 1.17 | 1.09 | 1.27 | 3.08 | 2.04 | 4.66 | 1.19 | 1.10 | 1.28 | 2.78 | 1.82 | 4.25 |
| Shortness of breath | 0.87 | 0.80 | 0.94 | 2.97 | 1.94 | 4.53 | 0.95 | 0.87 | 1.04 | 2.57 | 1.66 | 3.97 |
| Loss of smell | 0.82 | 0.75 | 0.90 | 3.90 | 2.55 | 5.96 | 0.93 | 0.85 | 1.03 | 3.72 | 2.37 | 5.85 |
| Loss of taste | 0.92 | 0.83 | 1.01 | 4.38 | 2.88 | 6.69 | 1.06 | 0.96 | 1.17 | 4.14 | 2.63 | 6.53 |
| Diarrhea | 1.00 | 0.70 | 1.42 | 1.45 | 0.20 | 10.51 | 0.83 | 0.58 | 1.19 | 1.13 | 0.15 | 8.48 |
| Contact with infected person | 0.65 | 0.61 | 0.70 | 4.40 | 2.87 | 6.75 | 0.76 | 0.70 | 0.82 | 5.16 | 3.18 | 8.36 |

Note: The multivariable regression model was adjusted for country, age, year, education, and user status with male users as reference group.

Supplementary Table 5. Proportion of reported COVID-19 related symptoms and contacts with an infected person stratified by education (n = 18,443)

|  | |  | | | Education | | | | |  | |  |
| --- | --- | --- | --- | --- | --- | --- | --- | --- | --- | --- | --- | --- |
| Variable | |  | ≤11 years  (n = 6,567) | | | ≥12 years  (n = 6,983) | | Missing  (n = 4,893) | | *P* | |  |
| Headache | % (n) | | | 27.2% (1,785) | | | 29.8% (2,084) | | 30.3% (1,482) | | <.001 | |
| Cough | % (n) | | | 26.8% (1,762) | | | 27.5% (1,920) | | 31.4% (1,534) | | <.001 | |
| General weakness | % (n) | | | 21.8% (1,431) | | | 24.8% (1,733) | | 29.2% (1,429) | | <.001 | |
| Muscle pain | % (n) | | | 20.6% (1,353) | | | 22.3% (1,559) | | 24.8% (1,213) | | <.001 | |
| Runny nose | % (n) | | | 20.9% (1,375) | | | 22.6% (1,575) | | 21.0% (1,026) | | .037 | |
| Fever | % (n) | | | 17.6% (1,159) | | | 19.1% (1,336) | | 27.0% (1,320) | | <.001 | |
| Sore throat | % (n) | | | 16.8% (1,106) | | | 21.7% (1,518) | | 20.9% (1,022) | | <.001 | |
| Shortness of breath | % (n) | | | 14.9% (976) | | | 15.0% (1,049) | | 16.9% (827) | | .005 | |
| Loss of smell | % (n) | | | 10.2% (670) | | | 11.5% (801) | | 15.4% (753) | | <.001 | |
| Loss of taste | % (n) | | | 9.7% (636) | | | 11.0% (767) | | 15.5% (758) | | <.001 | |
| Diarrhea | % (n) | | | 0.8% (54) | | | 0.8% (59) | | 0.5% (23) | | .038 | |
| Contact with infected person | % (n) | | | 23.9% (1,569) | | | 24.5% (1,710) | | 37.3% (1,826) | | <.001 | |

Supplementary Table 6. Unadjusted and adjusted odds ratios (ORs) stratified by education

|  | Unadjusted ORs | | | | | | Adjusted ORs | | | | | |
| --- | --- | --- | --- | --- | --- | --- | --- | --- | --- | --- | --- | --- |
|  | ≥12 years | | | missing | | | ≥12 years | | | missing | | |
| Variable | OR | 95% CI | | OR | 95% CI | | OR | 95% CI | | OR | 95% CI | |
| Headache | 1.14 | 1.06 | 1.23 | 1.16 | 1.07 | 1.26 | 1.13 | 1.05 | 1.22 | 1.07 | 0.98 | 1.17 |
| Cough | 1.03 | 0.96 | 1.12 | 1.25 | 1.15 | 1.35 | 1.02 | 0.94 | 1.10 | 1.03 | 0.95 | 1.13 |
| General weakness | 1.19 | 1.09 | 1.28 | 1.48 | 1.36 | 1.61 | 1.19 | 1.10 | 1.30 | 1.12 | 1.02 | 1.23 |
| Muscle pain | 1.11 | 1.02 | 1.20 | 1.27 | 1.16 | 1.39 | 1.09 | 1.00 | 1.19 | 1.05 | 0.96 | 1.15 |
| Runny nose | 1.10 | 1.01 | 1.19 | 1.00 | 0.92 | 1.10 | 1.14 | 1.05 | 1.24 | 1.00 | 0.91 | 1.10 |
| Fever | 1.10 | 1.01 | 1.20 | 1.72 | 1.58 | 1.89 | 1.01 | 0.91 | 1.11 | 1.01 | 0.91 | 1.12 |
| Sore throat | 1.37 | 1.26 | 1.50 | 1.30 | 1.19 | 1.43 | 1.39 | 1.27 | 1.51 | 1.18 | 1.06 | 1.30 |
| Shortness of breath | 1.01 | 0.92 | 1.11 | 1.17 | 1.05 | 1.29 | 1.00 | 0.90 | 1.10 | 0.90 | 0.81 | 1.01 |
| Loss of smell | 1.14 | 1.02 | 1.27 | 1.60 | 1.43 | 1.79 | 1.03 | 0.92 | 1.15 | 1.06 | 0.94 | 1.20 |
| Loss of taste | 1.15 | 1.03 | 1.29 | 1.71 | 1.53 | 1.91 | 1.03 | 0.92 | 1.15 | 1.11 | 0.99 | 1.25 |
| Diarrhea | 1.03 | 0.71 | 1.49 | 0.57 | 0.35 | 0.93 | 1.35 | 0.93 | 1.97 | 1.17 | 0.71 | 1.92 |
| Contact with infected person | 1.03 | 0.96 | 1.12 | 1.90 | 1.75 | 2.06 | 0.93 | 0.85 | 1.02 | 1.08 | 0.98 | 1.19 |

Note: The regression model was adjusted for country, gender, age, year, and user with ≤11 years of education as reference group.

Supplementary Table 7. Proportion of reported COVID-19 related symptoms and contacts with an infected person stratified by calendar year of using the app (n = 18,443)

|  |  | Year using App | |  |
| --- | --- | --- | --- | --- |
| Variable |  | 2020  (n = 15,983) | 2021/22  (n = 2,460) | *P* |
| Headache | % (n) | 28.6% (4,578) | 31.4% (773) | .005 |
| Cough | % (n) | 28.0% (4,473) | 30.2% (743) | .023 |
| General weakness | % (n) | 24.3% (3,882) | 28.9% (711) | <.001 |
| Muscle pain | % (n) | 22.1% (3,537) | 23.9% (588) | .050 |
| Runny nose | % (n) | 21.3% (3,412) | 22.9% (564) | .076 |
| Fever | % (n) | 20.5% (3,274) | 22.0% (541) | .086 |
| Sore throat | % (n) | 19.2% (3,067) | 23.5% (579) | <.001 |
| Shortness of breath | % (n) | 15.5% (2,437) | 15.4% (379) | .933 |
| Loss of smell | % (n) | 11.8% (1,889) | 13.6% (335) | .011 |
| Loss of taste | % (n) | 11.4% (1,829) | 13.5% (332) | .003 |
| Diarrhea | % (n) | 0.9% (136) | 0% (0) | <.001 |
| Contact with infected person | % (n) | 27.1% (4,334) | 31.3% (771) | <.001 |

Supplementary Table 8. Unadjusted and adjusted odds ratios (ORs) by calendar year of using the app

|  | Unadjusted ORs | | | Adjusted ORs | | |
| --- | --- | --- | --- | --- | --- | --- |
|  | 2021/22 vs. 2020 | | | 2021/22 vs. 2020 | | |
| Variable | OR | 95% CI | | OR | 95% CI | |
| Headache | 1.14 | 1.04 | 1.25 | 1.11 | 1.01 | 1.21 |
| Cough | 1.11 | 1.02 | 1.22 | 1.12 | 1.02 | 1.23 |
| General weakness | 1.27 | 1.15 | 1.39 | 1.33 | 1.20 | 1.46 |
| Muscle pain | 1.11 | 1.00 | 1.22 | 1.13 | 1.02 | 1.25 |
| Runny nose | 1.10 | 0.99 | 1.21 | 1.08 | 0.98 | 1.20 |
| Fever | 1.09 | 0.99 | 1.21 | 1.17 | 1.05 | 1.31 |
| Sore throat | 1.30 | 1.17 | 1.43 | 1.28 | 1.16 | 1.42 |
| Shortness of breath | 1.00 | 0.89 | 1.12 | 1.03 | 0.91 | 1.16 |
| Loss of smell | 1.18 | 1.04 | 1.33 | 1.21 | 1.06 | 1.37 |
| Loss of taste | 1.21 | 1.07 | 1.37 | 1.24 | 1.09 | 1.41 |
| Contact with infected person | 1.23 | 1.12 | 1.35 | 1.37 | 1.23 | 1.52 |

Note: The multivariable regression model was adjusted for country, gender, age, education, and user status with 2020 as reference group.

Supplementary Table 9. Proportion of reported COVID-19 related symptoms and contacts with an infected person stratified by user status (n = 18,443)

|  |  | User status | | | |  | |  |
| --- | --- | --- | --- | --- | --- | --- | --- | --- |
| Variable |  | myself  (n = 16,035) | | for others (n = 2,408) | | *P* | |  |
| Headache | % (n) | | 28.0% (4,485) | | 36.0% (866) | | <.001 | |
| Cough | % (n) | | 26.5% (4,252) | | 40.0% (964) | | <.001 | |
| General weakness | % (n) | | 23.4% (3,751) | | 35.0% (842) | | <.001 | |
| Muscle pain | % (n) | | 21.2% (3,396) | | 30.3% (729) | | <.001 | |
| Runny nose | % (n) | | 20.6% (3,307) | | 27.8% (669) | | <.001 | |
| Fever | % (n) | | 17.9% (2,887) | | 39.0% (938) | | <.001 | |
| Sore throat | % (n) | | 18.5% (2,968) | | 28.2% (678) | | <.001 | |
| Shortness of breath | % (n) | | 14.3% (2,300) | | 22.9% (552) | | <.001 | |
| Loss of smell | % (n) | | 10.8% (1,726) | | 20.7% (498) | | <.001 | |
| Loss of taste | % (n) | | 10.4% (1,668) | | 20.5% (493) | | <.001 | |
| Diarrhea | % (n) | | 0.8% (122) | | 0.6% (14) | | .337 | |
| Contact with infected person | % (n) | | 24.5% (3,934) | | 48.6% (1,171) | | <.001 | |

Supplementary Table 10. Unadjusted and adjusted odds ratios (ORs) stratified by user status

|  | Unadjusted ORs | | | Adjusted ORs | | | |
| --- | --- | --- | --- | --- | --- | --- | --- |
|  | for others vs. myself | | | for others vs. myself | | | |
| Variable | OR | 95% CI | | OR | 95% CI | | |
| Headache | 1.45 | 1.32 | 1.58 | 1.28 | 1.17 | 1.41 |  |
| Cough | 1.85 | 1.69 | 2.02 | 1.58 | 1.44 | 1.73 |  |
| General weakness | 1.76 | 1.61 | 1.93 | 1.37 | 1.24 | 1.51 |  |
| Muscle pain | 1.62 | 1.47 | 1.78 | 1.38 | 1.25 | 1.52 |  |
| Runny nose | 1.48 | 1.34 | 1.63 | 1.42 | 1.29 | 1.57 |  |
| Fever | 2.92 | 2.66 | 3.20 | 2.09 | 1.90 | 2.31 |  |
| Sore throat | 1.73 | 1.57 | 1.90 | 1.53 | 1.39 | 1.70 |  |
| Shortness of breath | 1.78 | 1.60 | 1.97 | 1.45 | 1.30 | 1.62 |  |
| Loss of smell | 2.16 | 1.94 | 2.41 | 1.59 | 1.42 | 1.79 |  |
| Loss of taste | 2.22 | 1.98 | 2.48 | 1.60 | 1.43 | 1.80 |  |
| Diarrhea | 0.76 | 0.44 | 1.33 | 1.26 | 0.71 | 2.24 |  |
| Contact with infected person | 2.91 | 2.67 | 3.18 | 2.06 | 1.87 | 2.27 |  |

Note: The multivariable regression model was adjusted for country, gender, age, year and education with reporting for oneself as reference group.
